# Genetic characterization of the complete genome of a highly divergent simian T-lymphotropic virus (STLV) type 3 from a wild *Cercopithecus mona *monkey

**DOI:** 10.1186/1742-4690-6-97

**Published:** 2009-10-27

**Authors:** David M Sintasath, Nathan D Wolfe, Hao Qiang Zheng, Matthew LeBreton, Martine Peeters, Ubald Tamoufe, Cyrille F Djoko, Joseph LD Diffo, Eitel Mpoudi-Ngole, Walid Heneine, William M Switzer

**Affiliations:** 1 Department of International Health, Johns Hopkins Bloomberg School of Public Health, Baltimore MD 21205, USA; 2 Global Viral Forecasting Initiative, San Francisco, CA, 94105, USA; 3 Stanford University, Program in Human Biology, Stanford, CA 94305, USA; 4 Laboratory Branch, Division of HIV/AIDS Prevention, National Center for HIV/AIDS, Viral Hepatitis, STD, and TB Prevention, Centers for Disease Control and Prevention, Atlanta, GA 30333, USA; 5 UMR 145, Institut de Recherche pour le Developement (IRD) and University of Montpellier 1, Montpellier, France; 6 Centre de Recherche du Service Santé des Armées (CRESAR), Yaoundé, Cameroon

## Abstract

**Background:**

The recent discoveries of novel human T-lymphotropic virus type 3 (HTLV-3) and highly divergent simian T-lymphotropic virus type 3 (STLV-3) subtype D viruses from two different monkey species in southern Cameroon suggest that the diversity and cross-species transmission of these retroviruses are much greater than currently appreciated.

**Results:**

We describe here the first full-length sequence of a highly divergent STLV-3d(Cmo8699AB) virus obtained by PCR-based genome walking using DNA from two dried blood spots (DBS) collected from a wild-caught *Cercopithecus mona *monkey. The genome of STLV-3d(Cmo8699AB) is 8913-bp long and shares only 77% identity to other PTLV-3s. Phylogenetic analyses using Bayesian and maximum likelihood inference clearly show that this highly divergent virus forms an independent lineage with high posterior probability and bootstrap support within the diversity of PTLV-3. Molecular dating of concatenated *gag-pol-env-tax *sequences inferred a divergence date of about 115,117 years ago for STLV-3d(Cmo8699AB) indicating an ancient origin for this newly identified lineage. Major structural, enzymatic, and regulatory gene regions of STLV-3d(Cmo8699AB) are intact and suggest viral replication and a predicted pathogenic potential comparable to other PTLV-3s.

**Conclusion:**

When taken together, the inferred ancient origin of STLV-3d(Cmo8699AB), the presence of this highly divergent virus in two primate species from the same geographical region, and the ease with which STLVs can be transmitted across species boundaries all suggest that STLV-3d may be more prevalent and widespread. Given the high human exposure to nonhuman primates in this region and the unknown pathogenicity of this divergent PTLV-3, increased surveillance and expanded prevention activities are necessary. Our ability to obtain the complete viral genome from DBS also highlights further the utility of this method for molecular-based epidemiologic studies.

## Background

Simian and human T-lymphotropic viruses (STLV and HTLV, respectively) are diverse deltaretroviruses now consisting of four broad primate T-lymphotropic virus (PTLV) groups. PTLV-1, PTLV-2 and PTLV-3 include human (HTLV-1, HTLV-2, and HTLV-3) and simian (STLV-1, STLV-2, and STLV-3) viruses, respectively [1-8]. To date, a total of three individuals from southern Cameroon with reported nonhuman primate (NHP) exposures were found to be infected with the recently identified HTLV-3 [1,7,8]. PTLV-4 consists of only HTLV-4 which was reported from one individual in Cameroon with known exposure to NHPs [7]. A simian counterpart of this virus has yet to be identified. Moreover, recent phylogenetic analyses of a highly divergent STLV-1-like virus from a captive *Macaca arctoides *suggest the possibility of a fifth group, tentatively referred to as PTLV-5 [9]. There is currently no evidence that STLV-5 has crossed into humans. These recent discoveries of novel HTLVs and STLVs suggest a greater diversity of PTLVs than is currently appreciated.

Both HTLV-1 and -2 have spread globally and are pathogenic human viruses [10-13]. HTLV-1 causes adult T-cell leukemia/lymphoma (ATL), HTLV-1 associated myelopathy/tropical spastic paraparesis (HAM/TSP), and other inflammatory diseases in less than 5% of those infected [2,11,13]. HTLV-2 is less pathogenic than HTLV-1, but has been associated with a neurologic disease similar to HAM/TSP [10,12]. The recently discovered HTLV-3 and HTLV-4 viruses have not yet been associated with any diseases, but molecular analyses of the full-length genomes have identified functional motifs important for viral expression and possibly oncogenesis [14,15].

STLVs have been identified in diverse Old World monkeys and apes. STLV-1 has been found in at least 20 different Old World primate species in Africa and Asia, and phylogenetic analysis shows that STLV-1s cluster by geography rather than by host species suggesting they are easily transmitted among NHPs [2,3,5,16,17]. There are currently seven recognized PTLV-1 subtypes (A to G) that are comprised of genetically related HTLV-1 and STLV-1 strains from different primate species. The close relatedness and clustering of the various HTLV-1s and STLV-1s into distinct subtypes suggests that at least seven independent cross-species transmission events formed the genetic diversity of HTLV-1. Currently STLV-2 is comprised of only two strains, STLV-2(PP1664) and STLV-2(PanP), both of which were identified in two different troops of captive bonobos (*Pan paniscus*) [6].

Like STLV-1, STLV-3 has a wide geographic distribution amongst NHPs in Africa [18-27]. Because of the phylogeographical clustering of STLV-3 into distinct clades, four separate molecular subtypes have been proposed: East African (subtype A), West and Central African (subtype B), and West African (subtype C and D) clades [21]. STLV-3 infection has been identified in captive Ethiopian gelada baboons (*Theropithecus gelada*) [27], wild sacred baboons (*Papio hamadryas*) [25], wild hybrid baboons (*P. hamadryas *X *P. anubis *hybrid) [25,27], and captive Eritrean hamadryas baboons (*P. hamadryas*) [19], which together comprise the STLV-3 East African (subtype A) clade. The STLV-3 West and Central African (subtype B) clade is made up strains found among Senegalese olive baboons (*P. papio*) [21], Cameroonian and Nigerian red-capped mangabeys (*Cercocebus torquatus torquatus*), and Cameroonian agile mangabeys (*Cercocebus agilis*) [18,22,23]. Somewhat divergent subtype B STLV-3s have also been recently identified in grey- cheeked mangabeys (*Lophocebus albigena*) and moustached monkeys (*Cercopithecus cephus*) in Cameroon although the phylogeny of these viruses was inferred using relatively short *tax *and LTR sequences [20,24]. That all three HTLV-3 strains which have been recently discovered in Cameroon [1,7,8] cluster within the STLV-3 subtype B clade is of phylogenetic significance. STLV-3 subtype C consists of divergent viruses found in Cameroonian spot-nosed guenons (*Cercopithecus nictitans*) though phylogenetic inference of this particular clade is limited by analysis of only very short *tax-rex *sequences [20,26]. Full-length genomes of STLV-3 subtype C are currently not available. More recently, we identified a highly divergent STLV-3 strain in Cameroon from two different primate species, *C. mona *(Cmo8699AB) and *C. nictitans *(Cni78676AB) [24]. Based on preliminary analysis of partial gene regions, these new STLVs formed a possible fourth STLV-3 lineage outside all PTLV-3 subtypes but within the diversity of the PTLV-3 group that we tentatively called STLV-3 subtype D [24]. Both STLV-3(Cmo8699AB) and STLV-3(Cni7867AB) share 99% sequence homology in the *pol*, *tax*, and LTR regions and cluster together with high bootstrap support within the STLV-3 subtype D clade [24]. Together, these findings demonstrate the broad range of NHP host species susceptible to STLV infection and that STLV diversity is driven more by phylogeography than by co-divergence with host species, illustrating the ease with which STLV is transmitted across species barriers [28,29].

Here, we report the first full-length genome sequence of STLV-3(Cmo8699AB) from a wild *C. mona *monkey. We confirm that this virus is a highly divergent and novel STLV-3. Across the genome, we found evidence that STLV-3d(Cmo8699AB) is unique from other PTLVs. Robust phylogenetic analysis of major gene regions of STLV-3d(Cmo8699AB) as well as new *tax *sequences from the divergent STLV-3d(Cni3034) and STLV-3d(Cni3038) viruses demonstrate that STLV-3d(Cmo8699AB) is a novel and ancient lineage outside the diversity of all known PTLV-3, thus strongly supporting its subtype D designation. Detailed examination of the complete genome predicted that all enzymatic, structural, and regulatory genes were intact. Viral replication and pathogenic potential shown or hypothesized for other PTLV-3s have yet to be determined [14,15,30]. Given the inferred ancient origin of STLV-3d(Cmo8699AB), its prevalence in two primate species from the same geographical region, and the documented propensity for STLVs to cross species boundaries, STLV-3d may be more widespread than currently realized. These results underscore an unknown public health concern for STLV-3d, particularly in a region with frequent exposure to NHPs through hunting and butchering.

## Methods

### DNA preparation and PCR-based genome walking

Using the NucliSens nucleic acid isolation kits (Biomérieux, Durham, NC) as previously described [24], nucleic acids were extracted from two dried blood spots (DBS) each collected by two different hunters from a wild-caught *C. mona *monkey (Cmo8699AB) and a *C. nictitans *monkey (Cni7867AB). Due to the limited DBS material available, we successfully maximized DNA yield through additional elution of nucleic acids from the silica beads with water. DNA from Cni3034 and Cni3038 were prepared from whole blood using the Qiagen DNA extraction protocol (Valencia, CA). DNA quality and yield were evaluated in a semi-quantitative PCR amplification of the β-actin gene as previously described [31,32] and confirmed with the QuantiT dsDNA HS Assay kit (Invitrogen, Carlsbad, CA). A minimum total input of 10 ng of DNA was used in each reaction mixture with standard PCR conditions. DNA preparation and PCR assays were performed in different laboratories specifically equipped for the processing and testing of only NHP samples according to established precautions to prevent contamination.

**Figure 1:**
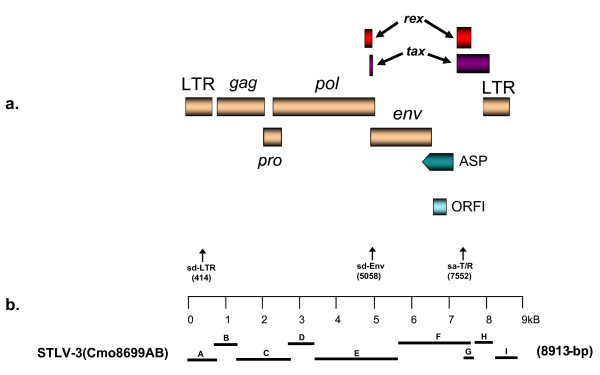
**STLV-3d(Cmo8699AB) genomic organization (a) and schematic representation of PCR-based genomic walking strategy (b)**. (a) Non-coding long terminal repeats (LTR), coding regions for all major proteins (*gag*, group specific antigen; *pro*, protease; *pol*, polymerase; *env*, envelope; *rex*, regulator of expression; *tax*, transactivator). (b) Short *tax *and LTR sequences (fragments A, G, H, and I) were amplified using generic primers as previously described [7,27,31]. Using a previously described PCR-based genomic walking strategy [14], the complete proviral sequence (8913-bp) was then obtained by using STLV-3d-specific primers located within each major gene region in combination with generic PTLV primers (fragments B - F). Amplicon sizes are approximated with the solid bars. The positions of predicted donor (sd) and acceptor (sa) splice sites are shown in parentheses.

Initially, small fragments of *tax *(222-bp) and *env *(371-bp) encoding regions of the STLV-3d(Cmo8699AB) genome were PCR-amplified using degenerate, nested primers, as previously described [14]. Using a PCR-based genome walking strategy, generic and STLV-3-specific primers were designed based on the short *tax *and *env *sequences, and the new STLV-3d(Cmo8699AB) or STLV-3d(Cni7867AB) sequences. Viral sequences > 2kb were then obtained using the Expand High Fidelity kit (Roche) following the manufacturer's protocol. For STLV-3d(Cmo8699AB), larger *tax *sequences (658-bp), overlapping sequences at the 3' end of *tax *to LTR (590-bp), and the remainder of the LTR (585-bp) were amplified using external and internal primers in standard PCR conditions as previously described [24]. Overlapping partial genomic fragments of the STLV-3d(Cmo8699AB) proviral genome and their expected amplicon sizes are shown in Fig. 1 and Table 1. Larger *tax *sequences (1047-bp) were generated for STLV-3c strains Cni3034 and Cni3038 using previously described forward outer and inner primers (PH1F and PH2F, respectively) [27] with the reverse outer, 8699LF4R (5'-TGG GTG GTT TAA GGT TTT TTC CGG-3') and inner primers, 8699LF3R (5'-ACA AGG CAG GGA GAG ACG TCA GAG-3'), respectively. STLV-3d(Cni7867AB) LTR-gag fragments (646-bp) were amplified using P5LF5 (5'-TCA ACC TTT TCT CCC CAA GCG CCT-3') and P3GR5 (5'-CYG CCT GRG CTA TGA GRG TCT CAA-3') as outer primer pairs and P5LF6 (5'-GCA CCT TCG CTT CTC CTG TCC TGG-3') and P3GR7 (5'-GRT AGG GYG GAG GCT TTT GRG GGT-3') as inner primers pairs. STLV-3d(Cni7867AB) pol-env fragments (2.3 kb) were amplified using outer primer pairs 7867GPF2 (5'-TCC ACA GAA AAA ACC CAA TCC ACT-3') and PGENVR1 [7] and 7867GPF3 (5'-CAC TCC TGG TCC CAT ACA CTT TCT CGG-3') and PGENVR2 [7] inner primer pairs. The nested primers 9589 F1 (5'-GGC CTR CTC CCG TGT CAR AAG GA-3') and 9589 R1 (5'-CCC AGG GTT CTT TAT TTG CTA GTC-3) and 9589 F2 (5'-ACC CCC GGG CTR ATT TGG ACT-3') and 9589 R2 (5'-GGC AAA CAT GAG GAA ATG GGT GGT-3') were used to amplify a 436-bp sequence from an STLV-3-infected *L. albigena *(Lal9589NL) to generate a 1,510-bp *tax*-LTR fragment using the *tax *and LTR sequences (GenBank accession numbers EU152289 and EU152277, respectively, obtained from this animal in another study [24].)

**Table 1:** PCR primer pairs^1,2 ^used to amplify overlapping regions of the STLV-3d(Cmo8699AB) genome

**Fragment**	**Region**	**Primer set**	**Primer**	**Sequence (5'-->3')**	**Primer**	**Sequence (5'-->3')**	**bp**
**B**	LTR-*gag*	Outer	P5LF5	TCA ACC TTT TCT CCC CAA CGC CCT	P3GR6	AYT GGR GGC TRC CWG GGG CGG AAG	954
		
		Inner	P5LF6	GCA CCT TCG CTT CTC CTG TCC TGG	P3GR7	GRT AGG GYG GAG GCT TTT GRG GGT	692

							

**C**	*gag-pol*	Outer	P5GF1	GTG CCG CCA ACC CCA TCC CCA AGG	PGPOLR1	GGY RTG IAR CCA RRC IAG KGG CCA	2687
		
		Inner	P5GF2	AAA GGG CTA GCA ATT CAC CAC TGG	P3GR1	GAT AGG GTT ATT GCC TGG TCC TTG ATA	1770

**D**	*pol*	Outer	8699GF20	ACC CCC CCA GTA AGC ATC CAG GCG	PGPOLR1	GGY RTG IAR CCA RRC IAG KGG CCA	1360
		
		Inner	8699GF21	AGA TGT CCT CCA GCA ATG CCA AAG	PGPOLR2	GRY RGG IGT ICC TTT IGA GAC CCA	992

							

**E**	*pol-env*	Outer	7867GPF2	TCC ACA GAA AAA ACC CAA TCC ACT	8699ETF2R	GGG CAG TAG CAA TGG GAC CAA GGA	2864
		
		Inner	7867GPF3	CAC TCC TGG TCC CAT ACA CTT TCT CGG	8699ETF1R	GGT GGG GCC TGT GTA GTT TGG GAG	2556

							

**F**	*env-tax*	Outer	7867EF1	AAA GTC TAA ACC CTC CAT GCC CAG	8699TR5	TTT GGT AGG GAT TTT TGT TAG GAA GG	2560
		
		Inner	7867EF2	TCC TTG TAT CTT TTT CCC CAT TGG	8699TR1	AAG GTA TTG TAG AGG CGA GCT GAC	2147

^1 ^The primers used to amplify *tax *and LTR overlapping regions (fragments A, G, H, I depicted in figure 1) are described elsewhere [24].

2. I = inosine; other letters are as defined by the IUPAC code.

PCR amplicons were purified with Qiaquick PCR or gel purification kits (QIAGEN, Valencia, CA) and sequenced directly using ABI PRISM Big Dye terminator kits (Foster City, CA) on an ABI 3130xl sequencer or after cloning into a TOPO vector (Invitrogen, Carlsbad, CA).

### Sequence and phylogenetic analysis and dating the origin of STLV-3d(Cmo8699AB)

Comparison of the full-length, gap-stripped PTLV-3 genomes was performed with the SimPlot program (Version 3.5.1) where STLV-3d(Cmo8699AB) was the query sequence using the F84 (ML) model and a transition/transversion ratio of 2.0 [33]. RNA secondary structure of the LTR region was predicted using the mfold web server program [34] found at . Prediction of splice acceptor (sa) and splice donor (sd) sites was performed using the NetGene2 program available at the web server [35]. Identification and analysis of ORFs were performed using the ORF Finder program available at .

Percent nucleotide divergence was calculated using the DNASTAR MegAlign 7.2 software (). For phylogenetic analysis two datasets were used. To investigate the phylogenetic relationship between PTLV, the first dataset included *tax *sequences from complete PTLV genomes available at GenBank and the new STLV-3 *tax *sequences from Cmo8699AB, Cni7867AB, Cni3034, Cni3038, and Lal9859 obtained in the current study, respectively. For further phylogenetic resolution of STLV-3d among PTLV, a larger dataset was used and included concatenated *gag*, *pol, env*, and *tax *sequences from complete PTLV genomes available at GenBank and the complete genome of STLV-3d(Cmo8699AB) determined here. Sequences were aligned using the Clustal W program, followed by manual editing and removal of indels. Nucleotide substitution saturation was assessed using pair-wise transition and transversion versus divergence plots using the DAMBE program [36]. Unequal nucleotide composition was measured by using the TREE-PUZZLE program [37]. Nucleotide substitution models and parameters were estimated from the edited Clustal W sequence alignments by using Modeltest v3.7 [38]. A variant of the general time reversible (GTR) model, which allows six different substitution rate categories (r_A ↔ C _= 2.62, r_A ↔ G _= 13.07, r_A ↔ T _= 2.79, r_C ↔ G _= 2.26, r_C ↔ T _= 4.54, r_G ↔ T _= 1) with gamma-distributed rate heterogeneity (α = 0.7071) and an estimated proportion of invariable sites (0.3436) was determined to best fit the data for the *tax *only alignments. The best model for the concatenated *gag-pol-env-tax *alignment was GTR+G, with six different rate substitutions (r_A ↔ C _= 2.53, r_A ↔ G _= 11.47, r_A ↔ T _= 2.58, r_C ↔ G _= 2.15, r_C ↔ T _= 4.3, r_G ↔ T _= 1) and gamma-distributed rate heterogeneity (α = 0.366). Phylogenetic trees were inferred using Bayesian analysis implemented in the BEAST software package [39] and with maximum likelihood (ML) using the PhyML program available online at the webserver [40]. Support for branching order of the ML-inferred trees was evaluated using 500 bootstraps. Two independent BEAST runs consisting of 10 - 100 million Markov Chain Monte Carlo (MCMC) generations for the *tax *only and PTLV concatamer alignments, respectively, with a sampling every 1,000 generations, an uncorrelated log-normal relaxed molecular clock, and a burn-in of 100,000 to 1 million generations. Both the constant coalescent and the Yule process of speciation were used as tree priors to infer the viral tree topologies. Convergence of the MCMC was assessed by calculating the effective sampling size (ESS) of the runs using the program Tracer (v1.4; ). All parameter estimates showed significant ESSs (> 300). The tree with the maximum product of the posterior clade probabilities (maximum clade credibility tree) was chosen from the posterior distribution of 9,001 sampled trees (after burning in the first 1,000 sampled trees) with the program TreeAnnotator version 1.4.6 included in the BEAST software package [40]. Trees were viewed and edited using FigTree v1.1.2 .

Divergence dates for the most recent common ancestor (MRCA) of STLV-3d(Cmo8699AB) were obtained by using both the *tax *only and the concatenated *gag-pol-env-tax *alignments, using Bayesian inference and using a relaxed molecular clock in the BEAST program. The PTLV evolutionary rate assumed a global molecular clock model and was estimated according to the formula: evolutionary rate (*r*) = branch length (*bl*)/divergence time (*t*) [27]. Divergence dates were obtained from well-established genetic and archaeological evidence for the timing of migration of the ancestors of indigenous Melanesians and Australians from Southeast Asia [14,16,29,41]. The PTLV evolutionary rate was estimated by using the divergence time of 40,000 - 60,000 years ago (ya) for the Melanesian HTLV-1 lineage (HTLV-1mel) and 15,000-30,000 ya for the most recent common ancestor of HTLV-2a/HTLV-2b native American strains as strong priors in a Bayesian MCMC relaxed molecular clock method implemented in the BEAST software package [39]. The use of two calibration points has previously been shown to provide more reliable estimates of PTLV substitution rates than a single calibration date [41,42]. The upper and lower divergence times estimated from anthropological data were used to define the interval of a strong uniform prior distribution from which the MCMC sampler would sample possible divergence times for the corresponding node in the tree.

### Nucleotide accession numbers

The STLV-3d(Cmo8699AB) complete proviral genome has the GenBank accession number EU231644. Partial STLV-3d genomic sequences obtained from monkey Cni7867AB were assigned the GenBank accession numbers FJ957879 (LTR-partial *gag*) and FJ957880 (*pol*-partial *env*). Longer *tax *sequences obtained from STLV-3d(Cni7867AB), STLV-3c(Cni3034), STLV-3c(Cni3038), and STLV-3b(Lal9589NL) have the GenBank accession numbers EU152281, FJ957877, FJ957878, and GQ241937, respectively.

## Results

### Comparison of the STLV-3d(Cmo8699AB) proviral genome with prototypical PTLVs

The complete STLV-3d(Cmo8699AB) proviral genome was obtained entirely from two DBS using a PCR-based genome walking approach to generate nine overlapping subgenomic fragments (Fig 1). The complete STLV-3d(Cmo8699AB) proviral genome was determined to be 8913-bp. Comparing the STLV-3d(Cmo8699AB) genome with other prototypical PTLVs suggests that this virus is highly divergent and has equidistant nucleotide identity from PTLV-1 (62%), PTLV-2 (64%), PTLV-4 (64%), and PTLV-5 (62%). Compared to the PTLV-3 group, STLV-3d(Cmo8699AB) has only 77% identity to prototypical HTLV-3s and STLV-3s (Table 2), sharing the highest nucleotide identity (77.3%) with HTLV-3(Pyl43). Complete genomes are not available for the recently reported STLV-3 subtype C sequences, Cni217 and Cni227 [26] and Cni3034 and Cni3038 [20] for comparison. However, we were able to generate longer *tax *sequences for STLV-3c(Cni3034; 1047-bp) and STLV-3c(Cni3038; 1048-bp), both of which shared 99% identity with each other and which shared 95% nucleotide identity with STLV-3d(Cmo8699AB) and about 83% identity with PTLV-3 subtypes A and B in this highly conserved region. Like STLV-3c and STLV3d subtypes, *tax *sequences from PTLV-3 subtypes A and B are very similar sharing about 92% nucleotide identity.

**Table 2:** Percent nucleotide and amino acid identity of STLV-3d(Cmo8699AB) with other prototypical PTLVs^1^

	**PTLV-3 (subtype A)**		**PTLV-3 (subtype B)**				
		
	**STLV-3** **(TGE-2117)**	**STLV-3 (PH969)**	**STLV-3 (CTO604)**	**STLV-3 (NG409)**	**STLV-3** **(PPA-F3)**	**HTLV-3** **(Pyl43)**	**HTLV-3 (2026ND)**
		
Genome	76.9	76.8	77.0	76.9	77.1	77.3	76.8
LTR	72.0	70.7	74.1	73.4	73.6	74.4	72.5
*gag*	79.6 (89.0)	78.9 (88.6)	79.6 (89.0)	79.2 (88.1)	79.9 (89.0)	79.6 (88.8)	78.6 (87.9)
p19	(87.0)	(88.0)	(87.9)	(85.9)	(87.0)	(87.9)	(87.0)
p24	(95.5)	(93.9)	(95.5)	(96.5)	(96.0)	(96.0)	(93.9)
p15	(83.1)	(83.1)	(83.1)	(80.7)	(81.9)	(80.2)	(83.1)
*pro*	70.9 (76.6)	72.2 (76.0)	73.1 (77.1)	72.7 (76.6)	72.0 (77.1)	72.4 (76.6)	73.3 (78.9)
*pol*	76.7 (82.3)	76.7 (82.7)	76.5 (82.0)	76.3 (82.2)	76.1 (82.5)	76.7 (82.2)	76.0 (80.9)
*env*	76.3 (84.3)	76.1 (83.1)	76.1 (83.2)	77.1 (84.9)	77.1 (85.1)	76.3 (83.6)	77.5 (84.9)
SU	(80.4)	(78.5)	(79.5)	(80.3)	(81.0)	(79.5)	(81.0)
TM	(91.5)	(91.5)	(89.8)	(90.9)	(92.6)	(90.9)	(92.0)
*rex*	89.1 (72.7)	88.7 (71.4)	87.7 (68.9)	88.5 (72.0)	87.9 (70.8)	87.9 (69.6)	87.2 (70.2)
*tax*	84.6 (90.2)	84.6 (88.8)	83.5 (89.1)	83.7 (89.1)	83.7 (88.8)	83.9 (89.7)	82.9 (87.6)

^1 ^Complete genomes were not available for STLV-3 subtype C viruses for comparison; amino acid identities are in parentheses.

**Figure 2:**
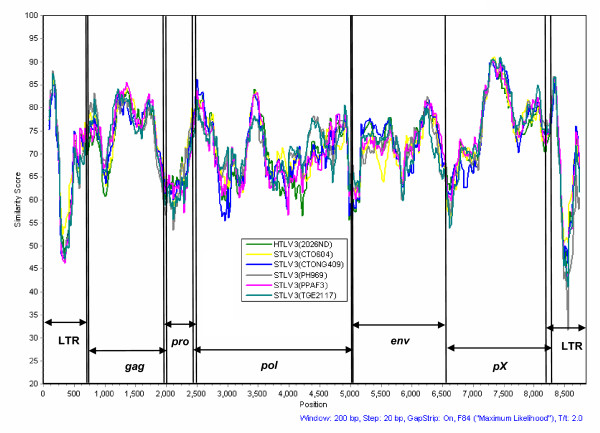
**Similarity plot analysis of the full-length STLV-3d(Cmo8699AB) and prototypical PTLV-3 genomes using a 200-bp window size in 20 step increments on gap-stripped sequences**. The F84 (maximum likelihood) model was used with an estimated transition-to-transversion ratio of 2.28. HTLV-3b(Pyl43) was not included in the analysis because of its high identity (> 99%) to STLV-3b(CTO604) and because of a 366-bp deletion in the pX region of this virus [15].

The predicted Tax and Gag proteins of STLV-3d(Cmo8699AB) were the most conserved proteins with the highest similarity (90 and 89%, respectively) to other prototypical PTLV-3 strains (Table 2). The highest genetic divergence between STLV-3d(Cmo8699AB) and other PTLV-3s was found in the non-coding LTR region (26-29%), and in the protease (Pro) (21-24%) and Rex (28 - 31%) proteins (Table 2). These genetic relationships are further illustrated in a similarity plot analysis comparing STLV-3d(Cmo8699AB) with other prototypical PTLV-3s across the entire genome (Fig. 2), where the highest and lowest sequence identities were observed in the *tax *and LTR regions, respectively.

### Evolutionary relationship of STLV-3d to other PTLVs

**Figure 3:**
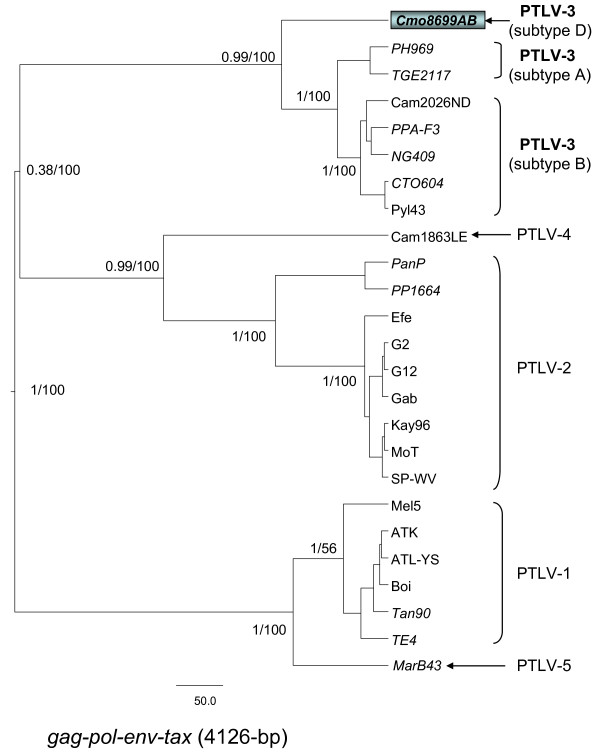
**Identification of a highly divergent STLV-3 subtype inferred by phylogenetic analyses of concatenated *gag-pol-env-tax *PTLV sequences (4,126-bp)**. First and second codon positions were used to generate PTLV phylogenies by sampling 10,000 trees with a Markov Chain Monte Carlo method under a relaxed clock model, and the maximum clade credibility tree, i.e. the tree with the maximum product of the posterior clade probabilities, is shown. Maximum likelihood trees were also inferred using the program PhyML and identical tree topologies were obtained with both methods. Posterior probabilities of inferred Bayesian topologies (numerator) and bootstrap support (1,000 replicates) for PhyML topologies (denominator) are provided at major nodes. The STLV-3d sequence reported here is shown boxed.

**Figure 4:**
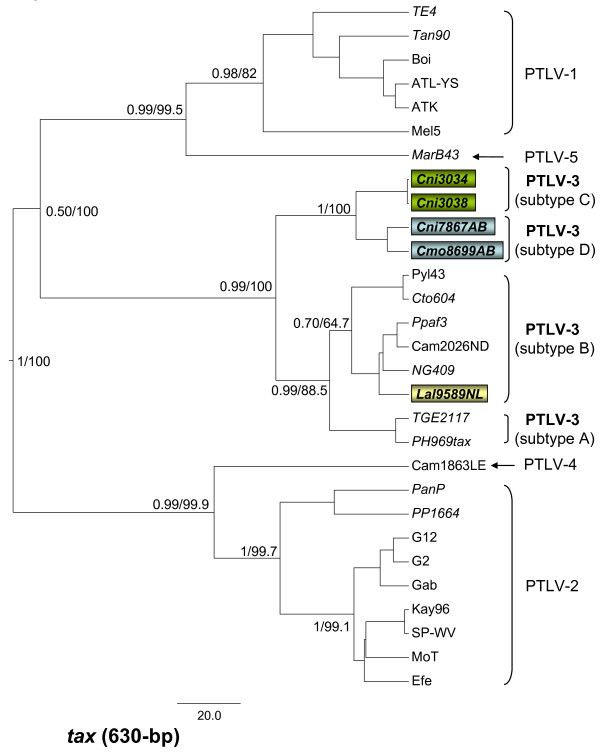
**Identification of a highly divergent STLV-3 subtype inferred by phylogenetic analyses of partial PTLV *tax *sequences (630-bp)**. First and second codon positions were used to generate PTLV phylogenies by sampling 10,000 trees with a Markov Chain Monte Carlo method under a relaxed clock model, and the maximum clade credibility tree, i.e. the tree with the maximum product of the posterior clade probabilities, is shown. Maximum likelihood trees were also inferred using the program PhyML and identical tree topologies were obtained with both methods. Posterior probabilities of inferred Bayesian topologies (numerator) and bootstrap support (1,000 replicates) for PhyML topologies (denominator) are provided at major nodes. STLV-3d and other new sequences generated in the current study from STLV-3c and STLV-3b-infected animals are boxed. Branch lengths are proportional to median divergence times in years estimated from the post-burn in trees with the scale at the bottom indicating 20,000 years.

Analysis of the two PTLV datasets for nucleotide substitution saturation using pair-wise transition and transversion versus divergence plots revealed that transitions and transversions plateaued at the 3^rd ^codon positions (cdp) indicating sequence saturation (data not shown) as previously observed [42]. In contrast, transitions and transversions increased linearly for the 1^st ^and 2^nd ^cdp without reaching a plateau indicating they still retained enough phylogenetic signal (data not shown). The BEAST and PhyML programs were then used to infer phylogenetic relationships of PTLV sequences using only 1^st ^and 2^nd ^cdp and the best-fit parameters defined above. The final nucleotide alignment lengths were 630-bp and 4126-bp for the *tax *only and viral concatamer sequences, respectively. Robust phylogenetic analysis of concatenated *gag-pol-env-tax *STLV-3d(Cmo8699AB) (Fig. 3) and *tax *sequences (Fig. 4) as well as sequences from other PTLV inferred a novel PTLV-3 subtype with very high posterior probabilities and bootstrap support. STLV-3d(Cmo8699AB) formed a distinct lineage from known PTLV-3 East African (subtype A) and West and Central African (subtype B) clades (Fig 3). Full-length genome sequences were not available for West African STLV-3c found in four *C. nictitans *or from STLV-3b sequences identified in *L. albigena *and *C. cephus *from Cameroon [20,26] for these analyses. However, phylogenetic analysis using longer *tax *sequences we obtained from two of these STLV-3 subtype C viruses (Cni3034 and Cni3038) and from a single *L. albigena *(Lal9859NL) indeed inferred a fourth distinct molecular subtype containing the STLV-3d(Cmo8699AB) and Cni7867AB *tax *sequences (Fig. 4). The new STLV-3(Lal9589NL) sequence clustered with other subtype B sequences from West-Central Africa (Fig. 4). Moreover, we identified another STLV-3 subtype D strain, STLV-3d(Cni7867AB) from a *C. nictitans *in the same geographic region that has 99% identity to STLV-3(Cmo8699AB) in the LTR-*gag*, *pol-env*, and *tax*-LTR regions and clusters tightly within the STLV-3 subtype D clade (Fig. 4). Combined, these results strongly support the identification and taxonomic classification of STLV-3(Cmo8699AB) and STLV-3(Cni7867AB) as a new PTLV-3 subtype. As has been shown before using individual genes, the phylogeny of the PTLV-3 clade in relation to PTLV-1, PTLV-2, and PTLV-4 was not completely resolved in the current Bayesian inference and clustered weakly with PTLV-2 and PTLV-4 using the *gag-pol-env-tax *concatamer and with PTLV-1 when using the *tax *only dataset (Figs. 3, 4).

### Divergence dates for the most recent common ancestor of STLV-3d(Cmo8699AB)

Additional molecular analyses were performed to estimate the divergence times of the MRCA of the potential new PTLV-3 subtype lineage using the 1^st ^and 2^nd ^cdp alignments and Bayesian inference and two independent fossil calibration points. The posterior mean evolutionary rate for PTLV was estimated to be 6.29 × 10^-7 ^and 5.36 × 10^-7 ^substitutions/site/year (Table 3) for the concatenated gene and the *tax *only alignments, respectively, which is consistent with rates determined previously both with and without enforcing a molecular clock [14,21-23,29,41]. The mean MRCA of STLV-3d(Cmo8699AB) is inferred to have split from PTLV-3a and PTLV-3b 115,117 ya (52,822 - 200,926 ya, 95% high posterior distribution (HPD)) based on the PTLV concatamer alignments (Table 3) suggesting that this is the oldest PTLV-3 lineage identified to date. Using the conserved *tax *only alignment STLV-3c and STLV-3d shared a common ancestor about 18,452 ya (4,386 - 36,666 ya 95% HPD) compared to 41,524 ya (17,149 - 68,097 ya 95% HPD) for divergence of STLV-3a and -b (Table 3). The inferred mean MRCA for the PTLV-3 group is 75,795 ya (33,342 - 127,209 ya 9% HPD) and 120,574 ya (52,894 - 201,260 ya 95% HPD) based on the *tax *only and PTLV concatamer alignments, respectively. The divergence dates for PTLV-3 inferred in the current analyses are higher than those reported previously because our analyses include the two new highly divergent STLV-3c and -d viruses which increase substantially the MRCA date for this clade. All other PTLV divergence dates are consistent with those obtained recently using 1^st ^and 2^nd ^cdp of individual PTLV genes, including the finding of lower divergence dates using only highly conserved *tax *genes [42].

**Table 3:** PTLV evolutionary rate and time-scale calculated with a Bayesian relaxed molecular clock using 1^st ^+ 2^nd ^codon positions of concatenated *gag-pol-env-tax *genes and *tax *only^1^.

**Clade**	***gag-pol-env-tax***	***tax*(630-bp)**
**Mean Posterior** **Substitution Rate^2^**	**6.29 × 10^-7^** **(3.29 × 10^-7 ^- 9.53 × 10^-7^)**	**5.36 × 10^-7^** **(3.21 × 10^-7 ^- 8.1 × 10^-7^)**

PTLV root	323,887 (147,042 - 529,980)	191,759 (88,914 - 299,436)
MarB43/PTLV-1	102,708 (58,833 - 109,552)	77,259 (45,899 - 118,645)
PTLV-1^**3**^	53,896 (38,355 - 76,651)	49,211 (39,783 - 59,155)
HTLV-4/PTLV-2	242,627 (77,653 - 305,591)	110,122 (46,324 - 180,712)
PTLV-2	107,191 (41,349 - 182,273)	67,460 (29,660 - 111,773)
STLV-2	42,350 (11,650 - 87,100)	31,018 (8,744 - 56,742)
HTLV-2	25,346 (14,419 - 40,104)	20,982 (13,591 - 27,792)
HTLV-2a, b^**4**^	21,492 (14,426 - 28,212)	20,947 (13,703 - 27,783)
PTLV-3	120,574 (52,894 - 201,260)	75,795 (34,342 - 127,209)
PTLV-3a/3b	54,953 (26,648 - 102,445)	41,524 (17,149 - 68,097)
**PTLV-3c/3d**	ND^5^	18,452 (4,386 - 36,666)
**PTLV-3d/3a+3b**	115,117 (52,822 - 200,926)	ND

1. The tMRCA is the median Bayesian estimate in years ago (ya); 95% HPD intervals are given in parentheses. ND = not determined.

2. Substitutions/site/year

3. The tMRCA for this node was constrained by using a uniform distribution prior of 40,000-60,000 ya.

4. The tMRCA for this node was constrained by using a uniform distribution prior of 15,000-30,000 ya.

5. The complete genome of STLV-3c is currently not available.

### Genomic organization of STLV-3d(Cmo8699AB) and identification of conserved functional motifs

**Figure 5:**
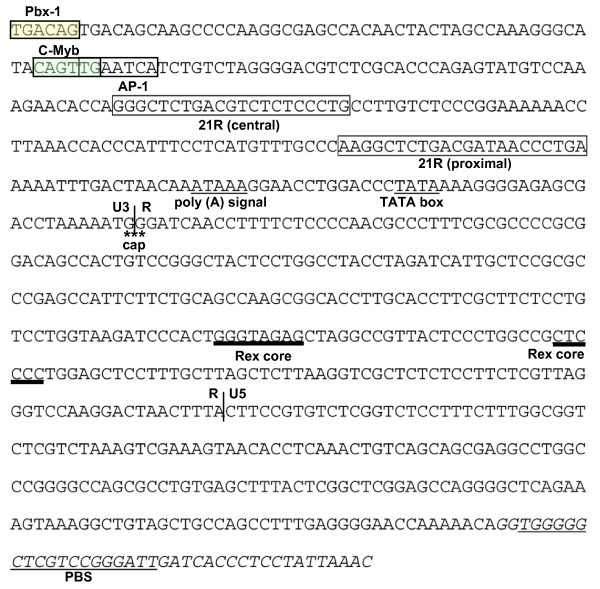
**Regulatory motifs identified in the nucleotide sequence of STLV-3d(Cmo8699AB) LTR and the pre-*gag *region:** various transcription factors are shown: pre-B cell leukemia (Pbx-1) and c-Myb (shaded); U3-R-U5 (vertical lines); AP-1 motif (boxed); approximate cap site (cap), polyadenylation [poly(A)] signal and TATA box (underlined); two 21-bp *tax*-responsive elements (21R) (boxed). In the R region, the predicted Rex core elements are underlined in bold. The pre-*gag *region and primer binding site (PBS) (underlined) are italicized.

**Figure 6:**
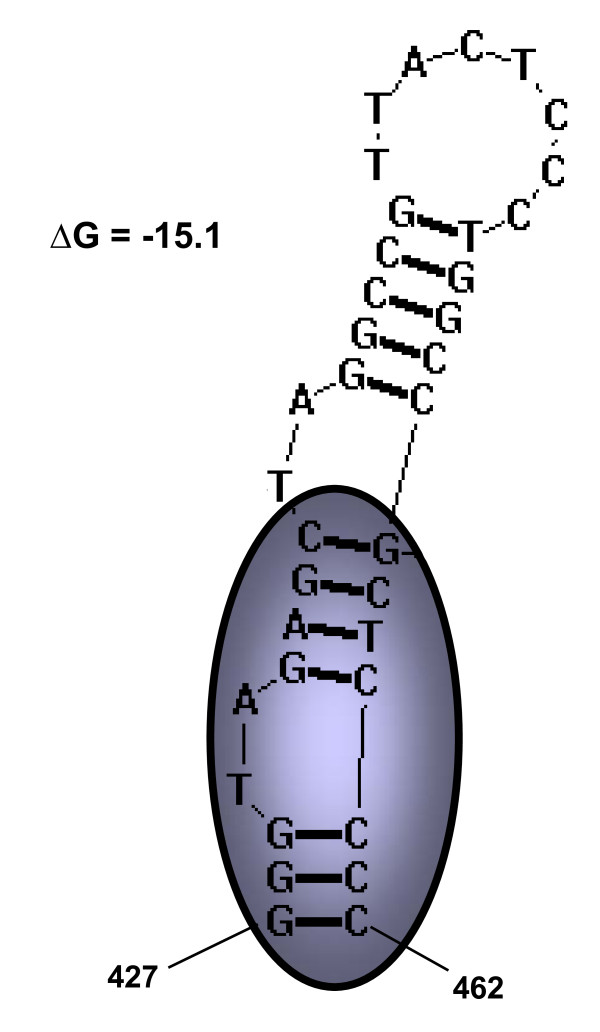
**Predicted stem-loop secondary structure of the STLV-3d(Cmo8699AB) LTR region showing the position of the Rex responsive element (RexRE) core (circled)**.

With regulatory and structural proteins flanked by long terminal repeats (LTRs) (Fig. 1), the genomic organization of STLV-3d(Cmo8699AB) resembles that of other prototypical replication competent PTLV strains. The STLV-3d(Cmo8699AB) LTR is 708-bp in length (Fig. 5) and as seen with other PTLV-3s, the STLV-3d(Cmo8699AB) LTR has two rather than the three highly conserved 21-bp tax-responsive element (TRE) repeat sequences found in HTLV-1 and HTLV-2 LTRs (Fig. 5). Conserved in all PTLV-3s, the cAMP-responsive element binding (CREB) motif (TGACGTC) [43] is present in the central TRE (nt 118 - 124) (where nt stands for nucleotide) (Fig. 5) that has been shown to be critical for binding of Tax and activation of gene expression [44]. Conserved regulatory motifs such as the polyadenylation signal (nt 221 - 225), TATA box (nt 239 - 242), cap site (nt 266 - 267), and splice donor site (nt 413 - 414) are all present in the STLV-3d(Cmo8699AB) LTR (Fig. 5). Similar to HTLV-3(Pyl43), STLV-3(PH969), STLV-3(TGE-2117) and STLV-3(CTO604), the activation protein-1 (AP-1) site is preserved in STLV-3d(Cmo8699AB) (Fig. 5). The conserved primer binding site (PBS) for PTLV, a 19-bp region between the 5' LTR and the *gag *gene and which allows reverse transcriptase to initiate synthesis of the viral DNA, is also present in STLV-3d(Cmo8699AB). Likewise, the heterogeneous nuclear ribonucleoprotein A1 (hnRNP-A1) binding site [TAG(G/A)(G/A)A] (nt 508 - 513), which has been suggested to play a critical role in RNA splicing and modulation of HTLV-1 gene expression [45], and the c-Myb (YAACKG) and pre-B-cell leukemia (Pbx-1, TGACAG) transcription factor binding sites associated with leukemogenesis [46] are all found in the LTR of STLV-3d(Cmo8699AB). The c-Myb and Pbx-1 sites are also present in the LTRs of STLV-3(CTO604), HTLV-3(Pyl43), STLV-2, and HTLV-4 [42]. The predicted RNA secondary structure of the STLV-3d(Cmo8699AB) LTR shows a stable stem-loop structure from nucleotides 427 - 462 containing the *rex *responsive element (RexRE) which plays a critical role in the polyadenylation of PTLV transcripts for viral gene expression (Fig. 6).

### STLV-3d proteome analysis

The predicted protein translation of the STLV-3d(Cmo8699AB) genome revealed all major structural and enzymatic (Gag, Pro, Pol, and Env) and regulatory proteins (Tax and Rex) (Fig. 1 and Table 2). Analysis of the overlapping open reading frames (ORFs) of *gag *and *pro *and *pro *and *pol *predicts that translation occurs by one or more successive -1 ribosomal frameshifts that align different ORFs. The conserved-slippage sequence (6(A)-8 nt-6(G)-11 nt-6(C) can be found in the *gag-pro *overlap of STLV-3d(Cmo8699AB). The *pro-pol *overlap slippage sequence has the same point mutation found among the other prototypical PTLV-3s (GTTAAAC versus TTTAAAC in HTLV-1, HTLV-2, and HTLV-4). Comparable to other PTLV-3s, the Gag protein of STLV-3d(Cmo8699AB) is composed of 420 amino acids (aa) and is predicted to cleave into three core protein products: p19 (matrix), p24 (capsid), and p15 (nucleocapsid). One of the most highly conserved PTLV domains, the Gag protein of STLV-3d(Cmo8699AB) has > 88% similarity to that of prototypical PTLV-3 subtypes (Table 2). The highest amino acid similarity to other PTLV-3 subtypes is found in the p24 capsid protein (94 - 96%), while the p15 nucleocapsid protein was the most divergent (80 - 83%).

The predicted length of the STLV-3d(Cmo8699AB) Env glycoprotein is 493 aa, similar to the Env protein of STLV-3b(CtoNG604) and HTLV-3b(Pyl43) (10, 35). The surface (SU) and transmembrane (TM) proteins are comparable to all other PTLV-3 subtypes at 315 aa and 178 aa, respectively. The TM protein is highly conserved across PTLV-3 subtypes (90 - 92% similarity) including STLV-3d(Cmo8699AB). The high aa identity of the Gag p24 and Env proteins suggests that this divergent virus would be cross-reactive on standard HTLV-1/2 Western blot (WB) assays. Unfortunately, serum or plasma was not available from animals Cmo8699AB and Cni7867AB to confirm this hypothesis. The STLV-3d(Cmo8699AB) SU also contains highly conserved residues believed important for viral entry (data not shown) similar to those described recently for HTLV-3b(Pyl43) [47].

PTLV Tax proteins are important for the trans-activation of viral gene expression, viral replication and viral pathogenesis. Comparison of the Tax proteins of prototypical PTLVs and STLV-3d revealed the conservation of critical functional motifs including the nuclear localization signal (NLS), cAMP response element (CREB) binding protein (CBP)/P300 binding motifs, and nuclear export signal (NES) motifs (data not shown). Amino acid sequences (M1, M22, and M47) that are important for Tax1 transactivation and activation of κβ (NF-κβ) pathway [48] are also preserved in the STLV-3d(Cmo8699AB) and STLV-3d(Cni7867AB) Tax proteins (data not shown). The C-terminal transcriptional activating domain (CR2) at positions 313 - 318 of the protein is important for CBP/P300 binding and up-regulation of transcription and is also present. The CR2 motif [(S/T)T(V/I)PFS] is conserved among all PTLV-3 subtypes and is identical to those found in STLV-3a subtypes. In the Tax C-terminus, STLV-3d also possesses a conserved PDZ-binding motif present in PTLV-1 and PTLV-3 Tax but not in PTLV-2 or HTLV-4 [14,30,42,49,50]. The PDZ domain has been shown to be an important binding site for Tax in mediating signal transduction and interleukin-2-independent growth induction for T-cell transformation [50,51]. Taken together, preservation of the predicted STLV-3d(Cmo8699AB) Tax protein sequence motifs suggests Tax interactions with cellular regulatory pathways similar to those of both PTLV-1 and PTLV-3. All functional motifs, including a potential PDZ domain, are present in the STLV-3d Tax, although 12 aa residues are missing from the N-terminus of the Tax proteins of STLV-3c(Cni3034 and Cni3038) obtained in the current study. This suggests that these motifs are highly conserved among the very divergent PTLV-3 group (data not shown).

While comparable in length to other PTLV-3s, the 182 aa Rex protein of STLV-3d(Cmo8699AB) is the most divergent viral protein sharing only about 70% similarity. The activation domain/NES (DALSARLYNTLSLDSPP) (aa 81 - 97) is an important motif conserved in all PTLVs for shuttling unspliced viral RNA transcripts from the nucleus to the cytoplasm [52,53]. However, like HTLV-2 and HTLV-4, the Rex protein of STLV-3d(Cmo8699AB) has an alanine (A) at position 94 instead of the glycine (G) found in HTLV-3b(Pyl43) and STLV-3b(CTONG409) or aspartic acid (D) present in all other PTLV-3s.

The pX encoding region between *env* and the 3' LTR contains multiple coding regions shown to be important for HTLV-1 viral replication T-cell activation, and cellular gene expression with two of the open reading frames (ORFs) encoding for the ubiquitous Tax and Rex proteins [54]. Two putative splice donor sites with high confidence were predicted at positions 414 and 5058 in the LTR (sd-LTR) and Env (sd-Env), respectively, that code for the Env protein (Fig. 1). A conserved splice acceptor site is located at position 7552 that with the sd-Env site code for the singly spliced Tax and Rex proteins (Fig. 1). The positions of these putative splice junction sites are similar to those of other PTLV-3s [14,15,21-23,27]. Analysis of the pX region of STLV-3d(Cmo8699AB) revealed only a single additional ORF (ORFI that begins with a methionine and is predicted to code for a proteins of 131 aa in length (Fig. 1)), in contrast to other PTLV-3s which have been predicted to have at least two additional ORFs in the pX region. BLAST analysis of the ORFI protein resulted in matches to miscellaneous fungal and mammalian proteins with very low identity (< 30%). Further studies are required to evaluate the function of the ORFI viral protein.

**Figure 7:**
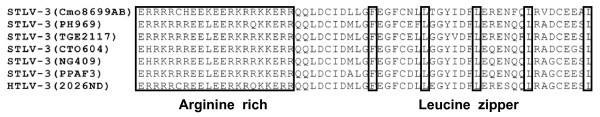
**Conservation of the antisense protein (ASP) of STLV-3d(Cmo8699AB) and other prototypical PTLV-3s**. Conserved arginine-rich region and potential leucine zipper motifs are indicated.

*In vivo *studies have demonstrated that the recently characterized basic leucine zipper (bZIP) factor found on the complementary minus-strand of the HTLV-1 RNA genome [55] can enhance viral infectivity and persistence [56]. Although originally discovered in HTLV-1 [55] and thus called the HTLV-1 bZIP (HBZ) protein, putative HBZ proteins have also been reported for all other PTLVs [14,15,42]. Consequently it has been proposed that HBZ be renamed as the HTLV antisense protein (ASP) [57]. As with other PTLVs, the ASP ORF of STLV-3d(Cmo8699AB) has a 21-aa arginine-rich region followed by 4 conserved leucine heptads and a leucine octet (Fig. 7), suggesting a similar inactivation pathway of cyclic AMP response element (CREB-2) and therefore, down-regulation of viral transcription [14,55]. Interestingly, the first "leucine" heptad in HTLV-1 and other PTLVs starts with another nonpolar amino acid: phenylalanine. This is unlike the leucine typically found in mammalian bZIP proteins. ASP has also been reported to modulate Tax activity by binding to the transcription factors JunB and c-Jun [58] as well as the ubiquitous AP-1 regulatory element [59]. The finding of an AP-1 site in the STLV-3d(Cmo8699AB) LTR may be a novel method for the regulation of viral transcription by ASP, as recently suggested for HTLV-3b(2026ND) [14]. Additional studies are necessary to validate and investigate a role for ASP in Tax expression and PTLV replication.

## Discussion

Screening of human populations with high exposure to NHPs has resulted in the successful discovery of novel retroviruses, including HTLV-3, HTLV-4, and simian foamy virus (SFV) [1,7,8,32]. We have previously demonstrated that hunter-collected DBS specimens from wild-caught NHPs are not only an effective collection strategy to demonstrate STLV diversity but also allow for monitoring of retroviral cross-species transmission events at the primate-hunter interface [24]. Using these primate DBS specimens, we recently identified novel STLV-3s in wild-caught *C. mona *and *C. nictitans *monkeys by analysis of partial gene sequences [24]. To characterize this new virus, we obtained its complete proviral genome using nucleic acids extracted entirely from two DBS, collected by two hunters in the field. To our knowledge, this is the first full-length genome of a simian retrovirus obtained entirely from DBS. The ability to generate a complete viral genome from the equivalent of about 0.25 ml whole blood demonstrates further the utility of this collection strategy for monitoring and characterizing viral diversity.

Robust phylogenetic analysis of both the conserved *tax *region and *gag-pol-env-tax *concatenated sequences inferred a novel lineage with high statistical support within the PTLV-3 clade that is highly divergent. The formation of a fourth lineage within the diversity of PTLV-3, containing STLV-3 sequences from two distinct primate species (*C. mona *and *C. nictitans*), strongly supports the proposed nomenclature and classification of this new virus as STLV-3 subtype D. The discovery of nearly identical STLV-3d(Cmo8699AB and Cni7867AB) viruses in two different primate species within the same region of Cameroon and the inferred ancient divergence of STLV-3d about 115,000 ya also suggests a higher prevalence and a more widespread distribution for this virus.

Our results also suggest that taxonomic subdivision within the current subtype B strains is warranted. PTLV-3 from Cameroon (STLV-3(Cto604), HTLV-3(Pyl43), and HTLV-3(Lobak18)) are distantly related to other PTLV-3 subtype B strains from West-Central Africa ((HTLV-3(Cam2026ND), STLV-3(NG409), STLV-3(PPAF3), and STLV-3(Lal9589NL) sharing < 90% nucleotide identity. Since both PTLV-3 lineages are presently known as B subtypes, we propose re-naming them as subtypes B1 (from Cameroon only) and B2 (from West-Central Africa). A similar nomenclature strategy has been previously adopted for subtyping HTLV-2 [60]. This re-classification and the categorization of the new STLV-3 subtype D are based upon highly supported phylogenetic division of these subtypes and genetic distances of at least 5% across the genome, as recently proposed for PTLV classification [42].

PTLVs have an ancient evolutionary history with the ancestral HTLVs being inferred to have first occurred many thousands of years ago following zoonotic transmission from STLV-infected NHPs [14,24,29,42,61]. This finding contrasts with the relatively recent emergence of the human immunodeficiency virus (HIV) from simian immunodeficiency virus-infected NHPs in the last century [62,63]. The recent discovery of HTLV-3 and HTLV-4 and novel STLV-1-like viruses among people who hunt and butcher NHPs suggests that these interspecies transmission events are not rare and are most likely contemporaneous [1,7]. From phylogenetic analysis it has been inferred that STLV-1 may have crossed species boundaries to humans on at least seven separate occasions resulting in the multiple HTLV-1 subtypes [28]. Given the inferred ancient origin of STLV-3d(Cmo8699AB) and PTLV-3, the wide geographic distribution of STLV-3 across Africa, the long history of human exposure to simians in Africa and the lack of screening for HTLV in blood banks in Africa, human infections with STLV-3d-like viruses might be expected to occur there. Thus, although HTLV-3 so far has only been identified in three persons from Cameroon and all three are subtype B viruses, it is tempting to speculate that like HTLV-1 diversity, HTLV-3 diversity will be driven by transmission of each of the four STLV-3 subtypes to humans. More surveillance studies at the NHP-human interface are needed to determine the prevalence, diversity, and epidemiology of STLV-3d(Cmo8699AB) and HTLV-3.

Molecular differences between HTLV-1 and HTLV-2 Tax proteins have been proposed to modulate function, transmissibility, and pathogenesis [61]. We therefore examined the predicted protein sequences of STLV-3d(Cmo8699AB) to determine whether important functional and regulatory motifs were present to infer the replication-competency and pathogenic potential for this divergent viral subtype. All enzymatic, structural, and regulatory proteins were preserved in STLV-3d(Cmo8699AB), including the ubiquitous Tax binding domains CBP/P300, NES, and CR2, which are all important for viral transcription and transformation [64-66]. In addition, the presence of a PDZ-binding motif in the STLV-3d Tax, which has been shown to be critical in signal transduction and T-cell transformation of HTLV-1-infected cells [50,51], suggests that the STLV-3d(Cmo8699AB) Tax is more similar to the Tax of PTLV-1 and other PTLV-3s, than it is to the Tax of PTLV-2 which lacks a PDZ motif [14,42]. Furthermore, as has been demonstrated with all PTLVs, STLV-3d also possesses a conserved ASP basic leucine zipper motif in the antisense strand between the *env *and *tax/rex *gene regions. ASP has been shown to participate in regulation of viral replication and possibly oncogenesis [50,51]. Combined, these findings show that the STLV-3d(Cmo8699AB) genome is intact, is likely to be replication competent, and may have a pathogenic potential similar to HTLV-1 which is also predicted for HTLV-3 subtype B; however, further studies are required to validate this hypothesis.

The LTR region of STLV-3d(Cmo8699AB) has two of the three 21-bp repeat Tax-responsive elements (TRE) typically found in the HTLV-1 and HTLV-2 LTRs. The three TREs (distal, central, and proximal) are involved in basal transcription and have been shown to confer Tax1, Tax2, and Tax3 responsiveness [67]. Studies have also shown that mutations in the central TRE compared to the distal or proximal TRE-1 result in the greatest loss of basal transcription levels [68]. As with all PTLV-3s, the STLV-3d(Cmo8699AB) LTR lacks only the distal TRE, which does not appear to have deleterious effects on gene expression and viral replication [30,69]. Nonetheless, more studies are necessary to determine if these differences will affect the transcriptional activity of STLV-3d(Cmo8699AB).

Another notable difference of STLV-3d from other PTLV-3s was observed in the leucine-rich activation region of the putative NES domain of the Rex protein involved in regulation of viral expression. STLV-3d(Cmo8699AB) has a single aa mutation from aspartic acid or glycine to alanine at position 94 similar to that seen in the HTLV-2 Rex protein (Rex2). Mutagenesis studies substituting alanine for serine residues in this region have demonstrated a significant reduction in the phosphorylation activation required for efficient RNA binding of Rex-2 [70]. These results suggest that the alanine mutation at aa position 94 of the STLV-3d(Cmo8699AB) Rex may also have a similar loss of biologic activity. The effects of these changes on the processing of viral transcripts and regulation of viral replication by the STLV-3d Rex will require further investigation.

## Conclusion

In summary, complete genome analysis of STLV-3d(Cmo8699) reveals this novel virus is a highly divergent member of the PTLV-3 group that we name subtype D. We show by robust genetic analysis that STLV-3d(Cmo8699AB) has an ancient origin and an intact genome. Furthermore, we demonstrate that complete viral genomes can be obtained using limited amounts of genomic material extracted from DBS collected in the field. This collection strategy will facilitate the monitoring of viral diversity and cross-species transmission at the human-primate interface. Expanded surveillance will help us to better understand the epidemiology and public health importance of STLV zoonoses.

## Competing interests

Some authors (WMS, NDW, DMS, WH) have applied for a patent for the discovery of STLV-3d.

## Authors' contributions

DMS obtained the full-length genome of STLV-3d, analyzed the sequences, and participated in writing the manuscript. WMS conceived, designed and coordinated the study, analyzed, acquired and interpreted the data, and wrote the manuscript. HZ obtained the *Lophocebus *STLV-3 sequences and helped write the manuscript. MP provided *C. nictitans *specimens and STLV-3 sequences and participated in writing the manuscript. ML, UT, JLDD, EMN, and NDW helped design the study, assisted in analysis of the data, and participated in writing the manuscript. All authors read and approved the final manuscript.
